# Effect of radiotherapy on cardiac-specific death in patients with non-malignant tumors of central nervous system and related clinical features

**DOI:** 10.3389/fcvm.2022.991621

**Published:** 2022-10-06

**Authors:** Ruxin Wang, Haowen Ye, Yongting Zhao, Li Ma, Jinjing Wei, Ying Wang, Xiaofang Zhang, Lihong Wang

**Affiliations:** ^1^Department of Endocrinology and Metabolism, The First Affiliated Hospital of Jinan University, Guangzhou, China; ^2^Department of Endocrinology, The Second Affiliated Hospital of Harbin Medical University, Harbin, China; ^3^Functional Examination Section, Gansu Provincial Maternity and Child-Care Hospital (Gansu Provincial Hospital), Lanzhou, China; ^4^Clinical Experimental Center, The First Affiliated Hospital of Jinan University, Guangzhou, China

**Keywords:** radiation therapy, non-malignant tumors, benign tumors, borderline tumors, central nervous system, cardiac-specific death

## Abstract

**Importance:**

Cardiac-specific death from radiation caused by radiation therapy (RT) in patients with malignant tumors has received extensive attention, however, little is known regarding the potential cardiotoxic effects of RT in patients with non-malignant tumors.

**Objectives and methods:**

In this study, we used the SEER data to explore the incidence of post-radiation cardiovascular complications in patients with non-malignant tumors of central nervous system (CNS), and identify the influencing factors of cardiac-specific death.

**Results:**

Ultimately 233, 306 patients were included (97.8% of patients had brain tumors and 2.2% had spinal cord tumors). For patients with non-malignant tumors of CNS, RT {yes (odds ratio [OR] 0.851, 95% confidence interval [CI] 0.774–0.936, *p* = 0.001, before propensity score matching (PSM); OR 0.792, 95% CI 0.702–0.894, *p* < 0.001, after PSM) vs. no} was associated with lower risk of cardiac-specific death, other clinical features affecting cardiac death similar to those in patients with non-malignant tumors of CNS receiving RT. For patients with non-malignant tumors of CNS receiving RT, female, married status, Hispanic ethnicity, surgery, and tumor site (brain exclude nerve and endocrine, nervous system) were associated with lower risks of cardiac-specific death, while earlier year of diagnosis, older age of diagnosis, Black, larger tumor and bilateral tumor were risk factors for cardiac-specific death.

**Conclusions:**

Our study shows the influencing factors for cardiac-specific death in patients with non-malignant tumors of CNS, and found RT is associated with lower risk of cardiac-specific death. These results can facilitate the identification of patients with non-malignant tumors of CNS who can benefit from RT while avoiding cardiovascular events. In addition, this study helps to enhance the clinical use of RT in these populations, especially in patients who may have impaired cardiac function due to CNS tumors.

## Introduction

Radiation therapy (RT) is the first line of treatment for specific cancers, and although effective against tumor cells, is also associated with multiple side effects. For instance, RT significantly increases the risk of cardiovascular complications and death (1), especially in patients with thoracic tumors and Hodgkin's lymphoma (2–4). Therefore, heart disease from radiation is a major concern in cancer therapy (5).

In addition to malignant tumors, non-malignant (benign and borderline malignancy) tumors have also received extensive attention. Non-malignant tumors growths are routinely treated by low-dose or medium-dose radiation (6). However, little is known regarding the potential cardiotoxic effects of RT in patients with non-malignant tumors (7), not to mention in patients with non-malignant tumors of central nervous system (CNS).

The National Cancer Institute's Surveillance, Epidemiology, and End Results (SEER) database is a population-based coordinated state cancer registry, which collates the demographic and clinical data of cancer patients from multiple regions across the United States (8). Since the SEER database also includes information on patients with non-malignant tumors (9), it is a potentially useful resource for exploring the additional cardiac effects of RT in this population (8, 9). In this study, we used the SEER data to explore the incidence of post-radiation cardiovascular complications in patients with non-malignant tumors of CNS, and identify the influencing factors of cardiac-specific death (10, 11).

## Patients and methods

### Data retrieval

The “Incidence” package of SEER database was accessed using SEER^*^Stat version 8.4.0.1, and data representing nearly 30% of the US population from 18 US registries collated between 2000 and 2019 was retrieved. The patients included in the analysis were 18 years of age and older with benign tumors or borderline malignancies (by selecting Behavior code ICD-O-3 = “Benign”, “Borderline malignancy” in the option of Site and Morphology), with or without RT. Cardiac-specific death included all heart diseases that led to death (corresponding variable in the SEER database is COD to site rec KM = “Diseases of Heart”), and patients were divided into non-cardiac and cardiac death groups. Ethical approval and patient consent were not required since the SEER data is publicly available (12).

### Statistical analysis

The missing data (Supplementary Table 1) were filled with multiple imputation, and variable (tumor size) not suitable for multiple imputation was imputed using expectation maximization. Categorical variables were expressed as frequencies and proportions. Pearson's chi-square test was used to compare variables with T (all theoretical numbers) ≥ 5 and n (total sample size) ≥ 40, continuity-corrected chi-square test was used if T ranged between ≥ 1 and < 5 and n was ≥ 40, and Fisher's test was used for variables with T < 1 or *n* < 40. Continuous variables that met normal distribution were expressed as mean ± SD, and compared by *t*-test if also homoscedastic. Otherwise, these variables were expressed as median (interquartile range [IQR]) and compared by Mann-Whitney *U* test. The factors significantly influencing cardiac-specific death were screened by univariate logistic regression, and used in multivariate analysis (“enter” method) to identify the independent risk factors. Sensitivity analysis of clinical features affecting cardiac-specific death was stratified by tumor nature and using logistic regression analysis. All statistical tests were two-sided, and *p* < 0.05 was considered statistically significant. SPSS (version, 27.0; SPSS, Chicago, Illinois, USA) was used for all statistical analyses.

### Propensity score matching

PSM is used in clinical research to improve comparability between groups in retrospective observational studies by adjusting for confounding factors (13). A propensity 1:1 matching analysis was performed using in this study by STATA (version, 17.0; STATA, Texas, USA) to minimize bias, followed by the statistical analyses as described above.

## Results

### Patient characteristics

A total of 233, 306 patients with non-malignant tumors of central nervous system (CNS) were included in the study. The average age of the patients at diagnosis was >55 years (cardiac-specific death group [76.33 ± 12.83]/no cardiac-specific death group [58.25 ± 17.44]), and the patients were predominantly White (80.9/78.5%), non-Hispanic ethnicity (92.1/85.6%), female (61.2/64.9%), married (3,791 [43.4%]/128, 455 [57.2%]), and middle-class (68.8/65.6%). The tumors were mainly located in the nervous system (78.8/66.8%). Less than half of patients underwent surgery (25.4/40.7%), a small percentage of patients had received RT (5.8/7.9%), and fewer patients were treated by chemotherapy (0.005/0.13%). There were significant differences between the cardiac and non-cardiac death groups except for the number of borderline tumors (Table 1). Patients with non-malignant tumors of CNS receiving RT had similar baseline characteristics to the patients with non-malignant tumors of CNS. The difference of characteristics between the cardiac and non-cardiac death groups were mainly in age of diagnosis, origin, marital status, year of diagnosis, and surgery (Table 2). In addition, cardiac-specific death was the leading cause of death among patients with non-malignant tumors of CNS receiving RT or not, with statistically different in incidence (RT [15.2%] vs. non-RT [17.5%], *p* = 0.001).

**Table 1:** Characteristics of patients included in non-malignant tumor patients.

**Variables**	**Before PSM**			**After PSM**		
	**Cardiac-specific death [*n* (%)]**	**No cardiac-specific death [*n* (%)]**	**P**	**Cardiac-specific death [*n* (%)]**	**No cardiac-specific death [*n* (%)]**	**P**
Number of patients (n)	8,741	224,565		8,740	8,740	
Age of diagnosis (years)	76.33 ± 12.83	58.25 ± 17.44	<0.001^*^	76.33 ± 12.82	76.16 ± 12.84	0.380
Race						
White	7,074 (80.9)	176,343 (78.5)	<0.001^*^	7,073 (80.9)	7,177 (82.1)	<0.001^*^
Black	1,105 (12.6)	26,454 (11.8)		1,105 (12.6)	818 (9.4)	
Asian or Pacific Islander	521 (6.0)	19,850 (8.8)		521 (6.0)	692 (7.9)	
American Indian	41 (0.5)	1,918 (0.9)		41 (0.5)	53 (0.6)	
Origin						
Hispanic ethnicity	692 (7.9)	32,229 (14.4)	<0.001^*^	692 (7.9)	886 (10.1)	<0.001^*^
Non-Hispanic ethnicity	8,049 (92.1)	192,336 (85.6)		8,048 (92.1)	7,854 (89.9)	
Sex						
Male	3,395 (38.8)	78,789 (35.1)	<0.001^*^	3,394 (38.8)	3,457 (39.6)	0.329
Female	5,346 (61.2)	145,776 (64.9)		5,346 (61.2)	5,283 (60.4)	
Marital status						
Non-married	4,950 (56.6)	96,110 (42.8)	<0.001^*^	4,949 (56.6)	4,328 (49.5)	<0.001^*^
Married	3,791 (43.4)	128,455 (57.2)		3,791 (43.4)	4,412 (50.5)	
Year of diagnosis	2,009.98 ± 4.089	2,012.42 ± 4.461	<0.001^*^	2,009.98 ± 4.09	2,009.88 ± 4.11	0.102
Chemotherapy						
No	8,737 (99.995)	224,266 (99.9)	0.026^*^	8,736 (100.0)	8,738 (100.0)	0.414
Yes	4 (0.005)	299 (0.1)		4 (0.0)	2 (0.0)	
Radiation						
No	8,232 (94.2)	206,905 (92.1)	<0.001^*^	8,231 (94.2)	8,092 (92.6)	<0.001^*^
Yes	509 (5.8)	17,660 (7.9)		509 (5.8)	648 (7.4)	
Biologica behavior						
Benign	8,235 (94.2)	207,841 (92.6)	<0.001^*^	8,234 (94.2)	8,173 (93.5)	0.055
Borderline malignancy	506 (5.8)	16,724 (7.4)		506 (5.8)	567 (6.5)	
Surgery						
No	6,519 (74.6)	133,064 (59.3)	<0.001^*^	6,518 (74.6)	6,437 (73.6)	0.162
Yes	2,222 (25.4)	91,501 (40.7)		2,222 (25.4)	2,303 (26.4)	
Income						
≤35,000	136 (1.6)	2,921 (1.3)	<0.001^*^	136 (1.6)	127 (1.5)	0.486
35,000–75,000	6,013 (68.8)	147,272 (65.6)		6,013 (68.8)	5,955 (68.1)	
≥75,000	2,592 (29.7)	74,372 (33.1)		2,591 (29.6)	2,658 (30.4)	
Tumor Site						
Brain exclude nerve and endocrine	376 (4.3)	13795 (6.14)	<0.001^*^	376 (4.3)	501 (5.7)	<0.001^*^
Nervous system	6,884 (78.8)	150,054 (66.82)		6,883 (78.8)	6,789 (77.7)	
Endocrine gland	1,481 (16.9)	60,704 (27.03)		1,481 (16.9)	1,450 (16.6)	
Other	0 (0)	12 (0.01)		0 (0)	0 (0)	
Tumor size	31 (17, 206)	58 (18, 216)	<0.001^*^	31 (17, 206)	30 (17, 203)	0.060
Number of benign borderline tumors						
1	8,305 (95.0)	214,333 (95.4)	0.058	8,305 (95.0)	8,329 (95.3)	0.398
>1	436 (5.0)	10,232 (4.6)		435 (5.0)	411 (4.7)	
Lateralityality						
Other	2,460 (28.1)	83,023 (37.0)	<0.001^*^	2,460 (28.5)	2,460 (28.1)	0.039^*^
Left	2,805 (32.1)	64,411 (28.7)		2,842 (32.5)	2,804 (32.1)	
Right	2,767 (31.7)	64,138 (28.6)		2,800 (32.0)	2,767 (31.7)	
Bilateral	709 (8.1)	12,993 (5.8)		608 (7.0)	709 (8.1)	

* *p* < 0.05.

**Table 2:** Characteristics of patients included in patients with non-malignant tumors of CNS receiving RT.

**Variables**	**Before PSM**			**After PSM**		
	**Cardiac-specific death [n (%)]**	**No cardiac-specific death [n (%)]**	**P**	**Cardiac-specific death [n (%)]**	**No cardiac-specific death [n (%)]**	**P**
Number of patients (n)	509	17,660		487	487	
Age of diagnosis (years)	71.65 ± 12.25	57.32 ± 14.51	<0.001^*^	71.04 + 12.15	72.39 + 13.49	0.102
Race						
White	405 (79.6)	13,946 (79.0)	0.002^*^	386 (79.3)	406 (83.4)	0.236
Black	69 (13.6)	1,752 (9.9)		68 (14.0)	50 (10.3)	
Asian or Pacific islander	32 (6.3)	1,843 (10.4)		30 (6.2)	30 (6.2)	
American Indian	3 (0.6)	119 (0.7)		3 (0.6)	1 (0.2)	
Origin						
Hispanic ethnicity	43 (8.4)	2,460 (13.9)	<0.001^*^	42 (8.6)	119 (24.4)	<0.001^*^
Non-Hispanic ethnicity	466 (91.6)	15,200 (86.1)		445 (91.4)	368 (75.6)	
Sex						
Male	236 (46.4)	6,851 (38.8)	0.001^*^	226 (46.4)	184 (37.8)	0.006^*^
Female	273 (53.6)	10,809 (61.2)		261 (53.6)	303 (62.2)	
Marital status						
Single	261 (51.3)	6,595 (37.3)	<0.001^*^	243 (49.9)	275 (56.5)	0.040^*^
Married	248 (48.7)	11,065 (62.7)		244 (50.1)	212 (43.5)	
Year of diagnosis						
2004–2007	240 (47.2)	3,955 (22.4)	<0.001^*^	218 (44.8)	191 (39.2)	0.052
2008–2011	166 (32.6)	4,511 (25.5)		166 (34.1)	162 (33.3)	
2012–2015	79 (15.5)	4,728 (26.8)		79 (16.9)	92 (18.9)	
2016–2019	24 (4.7)	4,466 (25.3)		24 (4.9)	42 (8.6)	
Chemotherapy						
No	508 (99.8)	17,557 (99.4)	0.254	486 (99.8)	485 (99.6)	0.563
Yes	1 (0.2)	103 (0.6)		1 (0.2)	2 (0.4)	
Biological behavior						
Benign	458 (90.0)	15,251 (86.4)	0.019^*^	436 (89.5)	428 (87.9)	0.418
Borderline malignancy	51 (10.0)	2,409 (13.6)		51 (10.5)	59 (12.1)	
Surgery						
No	362 (71.1)	10,815 (61.2)	<0.001^*^	342 (70.2)	289 (59.3)	<0.001^*^
Yes	147 (28.9)	6,845 (38.8)		145 (29.8)	198 (40.7)	
Income						
≤ 35,000	6 (1.2)	264 (1.5)	0.063	6 (1.2)	6 (1.2)	0.605
35,000–75,000	350 (68.8)	11,250 (63.7)		338 (69.4)	352 (72.3)	
≥75,000	153 (30.1)	6,146 (34.8)		143 (29.4)	129 (26.5)	
Tumor Site						
Brain exclude nerve and endocrine	20 (3.9)	695 (3.9)	0.201	19 (3.9)	17 (3.5)	0.608
Nervous system	410 (80.6)	14,776 (83.7)		392 (80.5)	404 (83.0)	
Endocrine gland	79 (15.5)	2,185 (12.4)		76 (15.6)	66 (13.6)	
Other	0 (0.0)	4 (0.0)				
Tumor size	29 (17, 85)	29 (18, 60)	0.902	32 (20,200)	35 (22,199)	0.241
Number of benign borderline tumors						
1	478 (93.9)	16,631 (94.2)	0.802	456 (93.6)	463 (95.1)	0.331
>1	31 (6.1)	1,029 (5.8)		31 (6.4)	24 (4.9)	
Lateralityality						
Left	176 (34.6)	6,607 (37.4)	0.062	167 (34.3)	175 (35.9)	0.678
Right	182 (35.8)	6,685 (37.9)		176 (36.1)	177 (36.3)	
Bilateral	35 (6.9)	889 (5.0)		35 (7.2)	26 (5.3)	
Other	116 (22.8)	3,479 (19.7)		109 (22.4)	109 (22.4)	

* *p* < 0.05.

**Figure 1:**
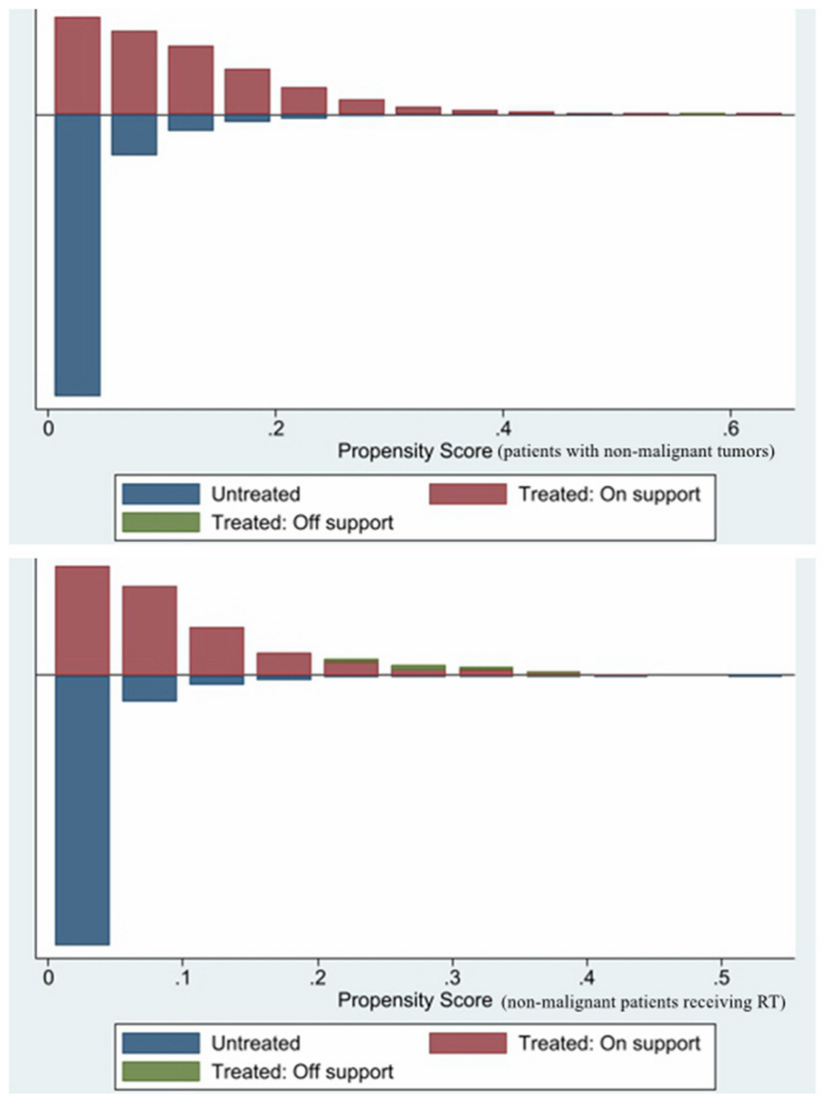
Distribution of propensity scores.

PSM was used to minimize the possible bias between the two groups. After 1:1 matching, 17, 480 patients with non-malignant tumors of CNS and 974 patients with non-malignant tumors of CNS receiving RT still retaining. Comparing the post-PSM with the pre-PSM, the differences in variables (age of diagnosis, sex, year of diagnosis, RT, biological behavior of tumor, surgery, income, tumor site, and tumor size) between the cardiac and non-cardiac death groups were significantly reduced in patients with non-malignant tumors of CNS (Table 1), the same is true for variables (age of diagnosis, race, marital status, year of diagnosis, and biological behavior of tumor) in patients with non-malignant tumors of CNS receiving RT (Table 2). Figure 1 shows distribution of propensity scores.

### Risk factors of cardiac-specific death in patients with non-malignant tumors of CNS

Multivariate logistic regression analysis showed that RT {yes (odds ratio [OR] 0.851, 95% confidence interval [CI] 0.774–0.936, *p* = 0.001, before PSM; OR 0.792, 95% CI 0.702–0.894, *p* < 0.001, after PSM) vs. no} was associated with lower risk of cardiac-specific death among patients with non-malignant tumors of CNS.

Besides, before PSM, race (White [OR 0.782, 95% CI: 0.730–0.839, *p* < 0.001], Asian or Pacific Islander [OR 0.600, 95% CI: 0.536–0.671, *p* < 0.001], American Indian [OR 0.649, 95% CI: 0.469–0.899, *p* = 0.009] vs. Black), origin (Hispanic ethnicity [OR 0.818, 95% CI: 0.753–1.888, *p* < 0.001] vs. non-Hispanic ethnicity), sex (female [OR 0.677, 95% CI: 0.643–0.713, *p* < 0.001] vs. male), marital status (married [OR 0.733, 95% CI: 0.698–0.769, *p* < 0.001] vs. non-married), year of diagnosis (OR 0.865, 95% CI: 0.861–0.870, *p* < 0.001), surgery (OR 0.870, 95% CI: 0.823–0.919, *p* < 0.001), higher income (≥75,000 [OR 0.692, 95% CI: 0.575–0.833, *p* < 0.001]; 35,000–75,000 [OR 0.794, 95% CI: 0.661–0.953, *p* = 0.013] vs. ≤35,000), and tumor site (brain exclude nerve and endocrine [OR 0.704, 95% CI: 0.610–0.812, *p* < 0.001] vs. endocrine gland) were associated with lower risks of cardiac-specific death among patients with non-malignant tumors of CNS, while age of diagnosis (OR 1.080, 95% CI: 1.078–1.082, *p* < 0.001) was a risk factor for cardiac-specific death (Table 3).

**Table 3:** Multivariate logistic regression analyses among patients with non-malignant tumors of CNS.

**Variables**	**Before PSM**			**After PSM**		
	**Multivariate analysis**			**Multivariate analysis**		
	**OR**	**95% CI**	**P**	**OR**	**95% CI**	**P**
Age of diagnosis (Years)	1.080	1.078–1.082	<0.001^*^			
Race						
Black		Reference			Reference	
White	0.782	0.730–0.839	<0.001^*^	0.788	0.714–0.869	<0.001^*^
Asian or Pacific islander	0.600	0.536–0.671	<0.001^*^	0.592	0.511–0.685	<0.001^*^
American Indian	0.649	0.469–0.899	0.009^*^	0.6	0.395–0.913	0.017^*^
Origin						
Non-Hispanic ethnicity		Reference			Reference	
Hispanic ethnicity	0.818	0.753–1.888	<0.001^*^	0.766	0.689–0.851	<0.001^*^
Sex						
Male		Reference				
Female	0.677	0.643–0.713	<0.001^*^			
Marital status						
Non-married		Reference			Reference	
Married	0.733	0.698–0.769	<0.001^*^	0.764	0.719–0.811	<0.001^*^
Year of diagnosis	0.865	0.861–0.870	<0.001^*^			
Chemotherapy						
No		Reference				
Yes	0.387	0.141–1.059	0.065			
Radiation						
No		Reference			Reference	
Yes	0.851	0.774–0.936	0.001^*^	0.792	0.702–0.894	<0.001^*^
Biological behavior						
Benign		Reference				
Borderline malignancy	0.998	0.892–1.117	0.978			
Surgery						
No		Reference				
Yes	0.870	0.823–0.919	<0.001^*^			
Income						
≤ 35,000		Reference				
35,000–75,000	0.794	0.661–0.953	0.013^*^			
≥75,000	0.692	0.575–0.833	<0.001^*^			
Site						
Endocrine gland		Reference			Reference	
Brain exclude nerve and endocrine	0.704	0.610–0.812	<0.001^*^	0.690	0.587–0.810	<0.001^*^
Nervous system	0.978	0.897–1.066	0.611	0.964	0.861–1.080	0.530
Other	0	0	0.999			
Tumor size	1.000	1.000–1.000	0.181	1.0001	1.0001–1.0002	<0.001^*^
Number of benign borderline tumors						
Laterality						
Left		Reference			Reference	
Right	0.981	0.928–1.038	0.512	1.002	0.93–1.079	0.964
Bilateral	1.076	0.984–1.177	0.109	1.133	1.003–1.280	0.044^*^
Other	0.991	0.917–1.072	0.825	0.995	0.898–1.102	0.92

* *p* < 0.05.

After PSM, race (White [OR 0.788, 95% CI: 0.714–0.869, *p* < 0.001], Asian or Pacific Islander [OR 0.592, 95% CI: 0.511–0.685, *p* < 0.001], American Indian [OR 0.6, 95% CI: 0.395–0.913, *p* = 0.017] vs. Black), origin (Hispanic ethnicity [OR 0.766, 95% CI: 0.689–0.851, *p* < 0.001] vs. non-Hispanic ethnicity), marital status (married [OR 0.764, 95% CI: 0.719–0.811, *p* < 0.001] vs. non-married), and tumor site (brain exclude nerve and endocrine [OR 0.690, 95% CI: 0.587–0.810, *p* < 0.001] vs. endocrine gland) were associated with lower risks of cardiac-specific death among patients with non-malignant tumors of CNS, while tumor size (OR 1.0001, 95% CI: 1.0001–1.0002, *p* < 0.001) and laterality (bilateral [OR 1.133, 95% CI: 1.003–1.280, *p* = 0.044] vs. left) were risk factors for cardiac-specific death (Table 3).

### Risk factors of cardiac-specific death in patients with non-malignant tumors receiving RT

**Figure 2:**
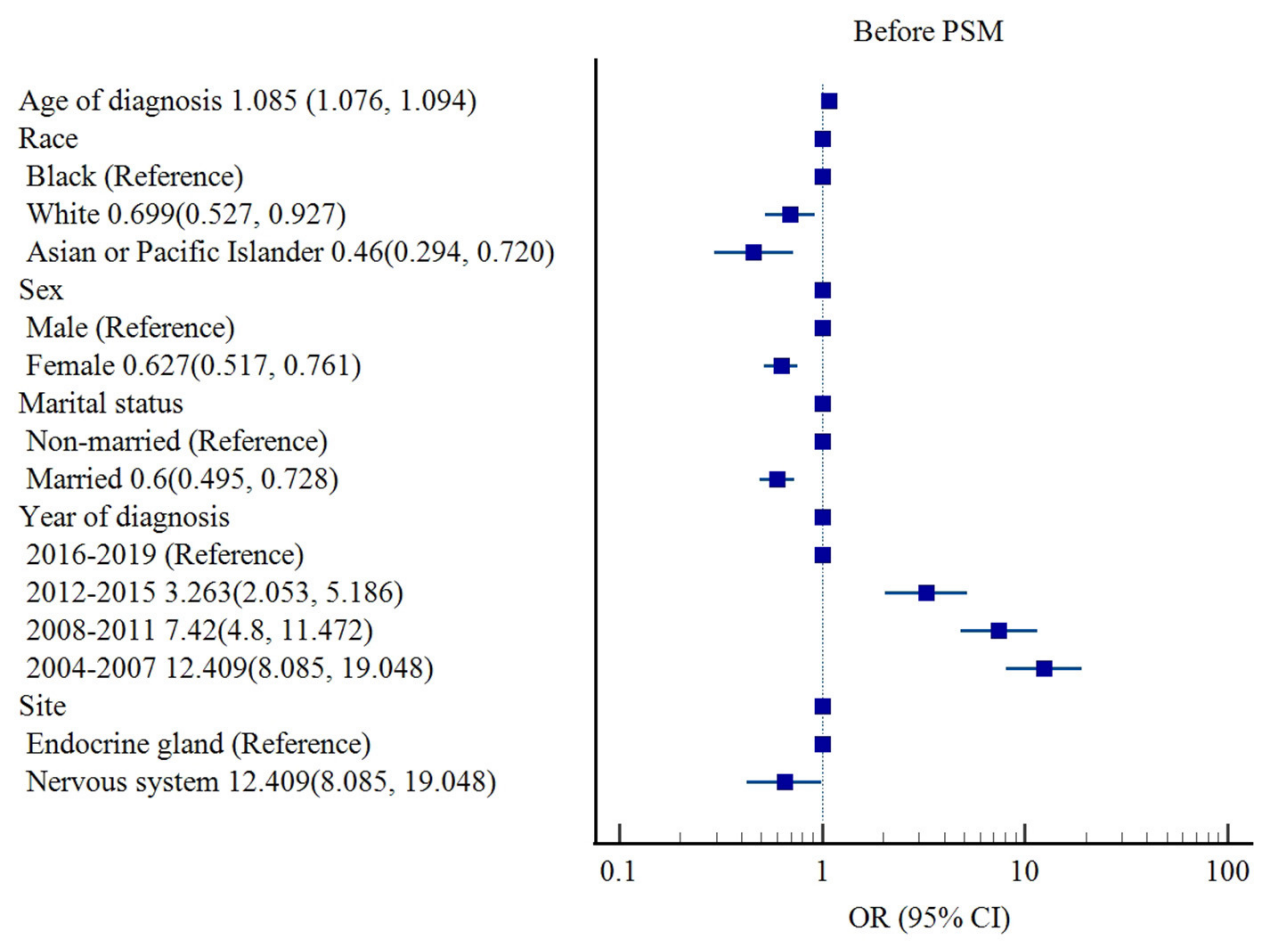
Influencing factors of cardiac-specific death in patients with non-malignant tumors of CNS receiving RT before PSM.

Before PSM, race (White [OR 0.699, 95% CI: 0.527–0.927, *p* = 0.013], Asian or Pacific Islander (OR 0.460, 95% CI: 0.294–0.720, *p* = 0.001] vs. Black), sex (female [OR 0.627, 95% CI: 0.517–0.761, *p* < 0.001] vs. male), marital status (married [OR 0.60, 95% CI: 0.495–0.728, *p* < 0.001] vs. non-married), and tumor site (nervous system [OR 0.650, 95% CI: 0.427–0.989, *p* = 0.044] vs. endocrine gland) were associated with lower risks of cardiac-specific death among patients with non-malignant tumors receiving RT, while older age of diagnosis (OR 1.085, 95% CI: 1.076–1.094, *p* < 0.001) and earlier year of diagnosis (2004–2007 [OR 12.409, 95% CI: 8.085–19.048, *p* < 0.001], 2008–2011 [OR 7.42, 95% CI: 4.8–11.472, *p* < 0.001], 2012–2015 [OR 3.263, 95% CI: 2.053–5.186, *p* < 0.001] vs. 2016-2019) were risk factors for cardiac-specific death (Table 4, Figure 2).

**Table 4:** Multivariate logistics regression analyses among patients with non-malignant tumors receiving RT.

	**Before PSM**			**After PSM**		
	**Multivariate analysis**			**Multivariate analysis**		
**Variables**	**OR**	**95% CI**	**P**	**OR**	**95% CI**	**P**
Age of diagnosis (Years)	1.085	1.076–1.094	<0.001^*^			
Race						
Black		Reference				
White	0.699	0.527–0.927	0.013^*^			
Asian or Pacific Islander	0.460	0.294–0.720	0.001^*^			
American Indian	1.270	0.380–4.249	0.698			
Origin						
Non-Hispanic ethnicity		Reference				
Hispanic ethnicity	0.844	0.606–1.176	0.317	0.289	0.197–0.424	<0.001^*^
Sex						
Male		Reference				
Female	0.627	0.517–0.761	<0.001^*^	0.726	0.552–0.955	0.022^*^
Marital status						
Non-married		Reference				
Married	0.600	0.495–0.728	<0.001^*^	1.190	0.907–1.561	0.209
Year of diagnosis						
2004–2007	12.409	8.085–19.048	<0.001^*^	1.952	1.122–3.394	0.018^*^
2008–2011	7.42	4.8–11.472	<0.001^*^	1.773	1.012–3.108	0.045^*^
2012–2015	3.263	2.053–5.186	<0.001^*^	1.435	0.787–2.619	0.239
2016–2019		Reference			Reference	
Chemotherapy						
No						
Yes						
Biological behavior						
Benign		Reference				
Borderline malignancy	1.069	0.752–1.519	0.709			
Surgery						
No		Reference				
Yes	1.107	0.870–1.408	0.401	0.584	0.443–0.768	<0.001^*^
Income						
≤35,000	0.825	0.354–1.923	0.655			
35,000–75,000	1.086	0.886–1.331	0.426			
≥75,000		Reference				
Site						
Endocrine gland		Reference				
Brain exclude nerve and endocrine	0.803	0.445–1.450	0.467			
Nervous system	0.650	0.427–0.989	0.044^*^			
Other	0.000	0.000	0.999			
Tumor size						
Number of benign borderline tumors						
Laterality						
Left		Reference				
Right	1.009	0.811–1.255	0.934			
Bilateral	1.379	0.932–2.040	0.108			
Other	0.854	0.588–1.242	0.410			

* *p* < 0.05.

**Figure 3:**
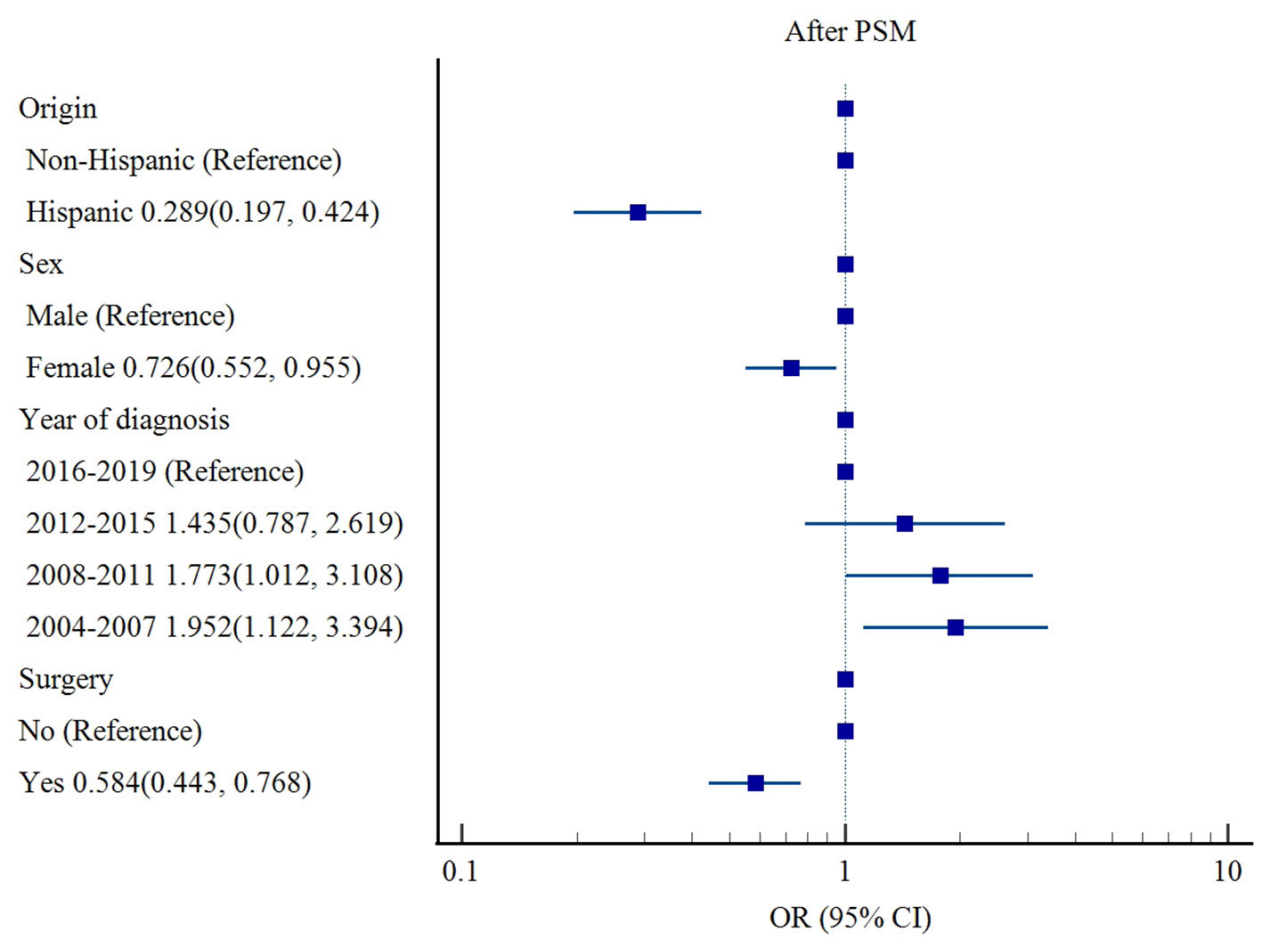
Influencing factors of cardiac-specific death in patients with non-malignant tumors of CNS receiving RT after PSM.

After PSM, origin (Hispanic ethnicity [OR 0.289, 95% CI: 0.197–0.424, *p* < 0.001] vs. non-Hispanic ethnicity), sex (female [OR 0.726, 95% CI: 0.552–0.955, *p* = 0.022] vs. male), and surgery (OR 0.584, 95% CI: 0.443–0.768, *p* < 0.001) were associated with lower risks of cardiac-specific death among patients with non-malignant tumors receiving RT, while earlier year of diagnosis (2004–2007 [OR 1.952, 95% CI: 1.122–3.394, *p* = 0.018], 2008–2011 [OR 1.773, 95% CI: 1.012–3.108, *p* = 0.045] vs. 2016–2019) was a risk factor for cardiac-specific death (Table 4, Figure 3).

### Sensitivity analysis

Clinical features affecting cardiac-specific death in patients with non-malignant tumors of CNS receiving RT were analyzed separately according to the nature of the tumor.

The results showed that among patients with benign tumors, before PSM, race (White [OR 0.715, 95% CI: 0.527–0.97, *p* = 0.031], Asian or Pacific Islander (OR 0.521, 95% CI: 0.33–0.824, *p* = 0.005] vs. Black) and sex (female [OR 0.62, 95% CI: 0.506–0.761, *p* < 0.001] vs. male) were associated with lower risks of cardiac-specific death, while older age of diagnosis (OR 1.089, 95% CI: 1.079–1.098, *p* < 0.001) and earlier year of diagnosis (2004–2007 [OR 12.695, 95% CI: 7.876–20.464, *p* < 0.001], 2008–2011 [OR 8.16, 95% CI: 5.03–13.238, *p* < 0.001], 2012–2015 [OR 3.766, 95% CI: 2.26–6.275, *p* < 0.001] vs. 2016–2019) were risk factors for cardiac-specific death. After PSM, origin (Hispanic ethnicity [OR 0.285, 95% CI: 0.188–0.43, *p* < 0.001] vs. non-Hispanic ethnicity), sex (female [OR 0.695, 95% CI: 0.523–0.923, *p* = 0.012] vs. male), and surgery (OR 0.536, 95% CI: 0.394–0.73, *p* < 0.001) were associated with lower risks of cardiac-specific death, while earlier year of diagnosis (2004-2007 [OR 2.118, 95% CI: 1.151–3.895, *p* = 0.016], 2008–2011 [OR 2.125, 95% CI: 1.145–3.943, *p* = 0.017] vs. 2016–2019) was a risk factor for cardiac-specific death.

Among patients with borderline malignancy tumors, before PSM, older age of diagnosis (OR 1.06, 95% CI: 1.038–1.083, *p* < 0.001) and earlier year of diagnosis (2004–2007 [OR 13.702, 95% CI: 5.107–36.758, *p* < 0.001], 2008–2011 [OR 4.419, 95% CI: 1.547–12.622, *p* = 0.006] vs. 2016–2019) were risk factors for cardiac-specific death. There was no statistically significant influences factor for cardiac-specific death after PSM.

## Discussion

Since the clinical benefits of RT for cancer therapy can be outweighed by RT-induced cardiotoxicity, the clinical importance of heart disease from radiation has been recognized by several major radiation oncology societies (14, 15). Meanwhile, it is crucial to explore strategies that can reduce the risk of heart disease from radiation (5). Previous studies have reported greater burden of cardiovascular risk factors and increased incidence of cardiovascular disease in patients with head or neck malignancies after undergoing RT (e.g., heart failure [hazard ratio 1.44, 95% CI: 1.18–1.76, *p* = 0.0005, coronary artery disease/myocardial infarction [hazard ratio 1.52, 95% CI: 1.03–2.24, *p* = 0.0357) (16–18). However, above situation may not need to be considered too much in patients receiving RT for non-malignant tumors of CNS, because our findings indicate that RT is associated with lower risk of cardiac-specific death in patients with non-malignant tumors of CNS. Furthermore, the cardiac-specific mortality was lower in patients that receive RT compared to the non-RT patients among patients with non-malignant tumors of CNS (2.8 vs. 3.8%, *p* < 0.001).

The parameters and outcomes of RT differ considerably between malignant and non-malignant tumors. Firstly, the dose and duration of RT are generally less for non-malignant tumors (19). Secondly, apart from the relatively high incidence of radiation-induced heart failure and a similar number of radiation-induced secondary cancers, primary malignant tumors are prone to post-radiation recurrence that may affect cardiac function through various ways (5). On the other hand, recurrence of non-malignant tumors after radiotherapy is less frequent. Finally, patients with non-malignant tumors rarely undergo chemotherapy, and therefore avoid the potential cardiotoxic effects of drugs. Altogether, the above factors may lead to different additive effects of RT in different nature of tumors. The growth and expansion of benign tumors compress the surrounding organs and constrict blood vessels, which can lead to dysfunction in various organs, including the brain. Natelson proposed the concept of a neuro-cardiological axis in 1985, which is based on the complex interplay between the nervous system of the brain and the cardiovascular system (20–22). For example, the CNS controls cardiovascular function through sympathetic and parasympathetic signals, and lesions in the cranial nervous can impair the structure and function of the cardiovascular system (23), ranging from myocardial damage to even cardiac death (24). Therefore, the cardio protective effect of RT observed in our study can be attributed to the reduction in tumor growth, which in turn minimized the risk of brain dysfunction and the ensuing brain-heart syndrome. Unlike chest irradiation, which elicited more cardiovascular events on the left side than on the right side (25, 26), patients with non-malignant tumors of CNS on the right side of head had no reduction in cardiac-specific death compared to those with tumors on the left side. However, bilateral tumors increased the risk of cardiac deaths. Further study is need to elucidate the mechanisms underlying this phenomenon.

Cancer patients have a consistently higher risk of death due to CVD, compared to the general population, and the risk increases with age (27). Furthermore, younger age at treatment, longer follow-up, and cardiac exposure also increase the risk of fatal cardiovascular diseases (5). In our study, older age of diagnosis and earlier year of diagnosis were risk factors for cardiac-specific death in patients with non-malignant tumors of CNS receiving RT. Furthermore, cardiovascular mortality is usually higher among patients with African ancestry, and lower among the Caucasian, Asian and Hispanic populations (28–31). Consistent with this, we found that Caucasian, Asian or Pacific Islander ethnicities were associated with lower risks of post-radiation cardiac-specific death in patients with non-malignant tumors of CNS receiving RT. According to the European Society of Cardiology, lower socioeconomic status may continue to contribute to increased the rate of cancer and the risk of death from cardiovascular diseases in cancer survivors (32). However, we found that the income level had no significant impact on the incidence of cardiac-specific death in patients with non-malignant tumors of CNS receiving RT. Other protective factors of radiation-induced cardiac-specific death in our cohort were the female (33) and married status. Marital stress, or single/divorced status increased the risk of cardiovascular death (34, 35). However, tumor size, number of benign borderline tumors, tumor site, and the biological behavior of tumor did not significantly affect cardiac-specific death in patients with non-malignant tumors of CNS receiving RT.

To further control for confounding factors and improve comparability between groups for more robust results, we matched the patients' baseline data using PSM (1:1) and then reanalyzed the matched data. Hispanic ethnicity and surgery were associated with lower risks of cardiac-specific death in patients with non-malignant tumors of CNS receiving RT. The differences in these results may be attributed to the smaller subset of patients after PSM, although the data was more comparable and the results more credible. Despite its advantages, PSM cannot fully represent the total population, Therefore, it is necessary to evaluate the pre- and post-PSM results for clinical studies. In addition, sensitivity analysis showed that in patients with benign tumors of CNS receiving RT, clinical features affecting cardiac-specific death were consistent with the overall results. However, in patients with borderline malignancy tumors of CNS, only age of diagnosis and year of diagnosis were influential factors for cardiac-specific death. This may be due to the small number (7.3%) of borderline malignancy tumors, which has a smaller contribution on the overall study results. Therefore, our findings are primarily reflective the situation in benign tumors. Overall, in patients with non-malignant tumors of CNS receiving RT, most factors affecting cardiac death were similar to those in patients with malignancies, except for some differences.

Factors affecting cardiac-specific death in patients with non-malignant tumors of CNS were similar to those in patients with non-malignant tumors of CNS receiving RT, except for married, earlier year of diagnosis, and tumor site (brain exclude nerve and endocrine) were consistently associated with lower risks of cardiac-specific death, lower income and larger tumor were risk factors for cardiac-specific death.

## Limitation

Although this is the first study analyzing the additional effect of RT on cardiovascular risks in patients with non-malignant tumors of CNS, it has some limitations that ought to be considered. Firstly, since we used SEER data that has been collated from different sources, the information may have been incomplete and some cardiac deaths may have been classified as unexplained deaths because they are not clear. Secondly, cardiac-specific death. Secondly, cardiac-specific deaths may be associated with other confounding factors that were not studied or not included in the SEER database. Thirdly, as a large number of patients did not experience cardiac-specific death, thus preventing the use of COX proportional hazards mode (censored values over 90%). Finally, the retrospective design of the study invariably led to bias.

## Conclusion

RT is associated with lower risk of cardiac-specific death in patients with non-malignant tumors of CNS, which indicates that the effect of radiation on the heart depends on the biological behavior of tumor. Hispanic ethnicity, female, married status, surgery and tumor site (nervous system) were the protective factors, whereas Black, older age of diagnosis and earlier year of diagnosis were risk factors for cardiac-specific death. These results can facilitate the identification of patients with non-malignant tumors of CNS who can benefit from RT while avoiding cardiovascular events. In addition, this study helps to enhance the clinical use of RT in these populations, especially in patients who may have impaired cardiac function due to CNS tumors.

## Data availability statement

The raw data supporting the conclusions of this article will be made available by the authors, without undue reservation.

## Ethics statement

Ethical review and approval was not required for the study on human participants in accordance with the local legislation and institutional requirements. Written informed consent for participation was not required for this study in accordance with the national legislation and the institutional requirements.

## Author contributions

RW and HY contributed to the conception and design of the study. LW and XZ provided administrative support. RW and YZ contributed to the collection and assembly of data. LM, YW, and JW did the literature search and applied the inclusion and exclusion criteria. RW, HY, and YZ analyzed and interpreted the data. RW, LW, and XZ contributed to the writing of the report. All authors approved the final version of the report.

## Funding

This study was supported by the Talent Introduction Funding Project of The First Affiliated Hospital of Jinan University (No. 808026) and Basic Scientific Research Project of Central University of Jinan University (No. 2162301).

## Conflict of interest

The authors declare that the research was conducted in the absence of any commercial or financial relationships that could be construed as a potential conflict of interest.

## Publisher's note

All claims expressed in this article are solely those of the authors and do not necessarily represent those of their affiliated organizations, or those of the publisher, the editors and the reviewers. Any product that may be evaluated in this article, or claim that may be made by its manufacturer, is not guaranteed or endorsed by the publisher.
