# Supplementary material for: Effect of radiotherapy on cardiac-specific death in patients with non-malignant tumors of central nervous system and related clinical features
Source: Front Cardiovasc Med. 2022 Oct 6;9:991621. doi: 10.3389/fcvm.2022.991621 (PMC9582928; doi:10.3389/fcvm.2022.991621)
Supplement: Supplementary file 1 [file Data_Sheet_1.docx]

Supplementary Table 1 Missing data in the original data.

| Variables | Missing data n (%) |
| --- | --- |
| Race | 3588 (1.5) |
| Surgery | 3568 (1.5) |
| Marital status | 16800 (7.2) |
| Income | 53 (0.02) |
| Tumor size | 70667 (30.2) |
| Number of benign borderline tumors | 79 (0.03) |
| Survival months | 1322 (0.5) |

.

Supplementary Table 2  Univariate logistics regression analyses among patients with non-malignant patients of CNS.

| Variables | Before Matching | | | After Matching | | |
| --- | --- | --- | --- | --- | --- | --- |
|  | Univariate analysis | | | Univariate analysis | | |
|  | OR | 95% CI | P | OR | 95% CI | P |
| Age of diagnosis (Years) | 1.08 | 1.078-1.081 | <0.001 | 1.001 | 0.999-1.003 | 0.380 |
| Race |  |  |  |  |  |  |
| Black |  | Reference |  |  | Reference |  |
| White | 0.960 | 0.900-1.025 | 0.221 | 0.730 | 0.663-0.803 | <0.001 |
| Asian or Pacific Islander | 0.628 | 0.565-0.698 | <0.001 | 0.557 | 0.482-0.644 | <0.001 |
| American Indian | 0.512 | 0.373-0.701 | <0.001 | 0.573 | 0.377-0.869 | 0.573 |
| Origin |  |  |  |  |  |  |
| Non-Hispanic |  | Reference |  |  | Reference |  |
| Hispanic | 0.513 | 0.474-0.555 | <0.001 | **0.762** | 0.687-0.846 | <0.001 |
| Sex |  |  |  |  |  |  |
| Male |  | Reference |  |  | Reference |  |
| Female | 0.851 | 0.815-0.889 | <0.001 | 1.031 | 0.970-1.095 | 0.329 |
| Marital status |  |  |  |  |  |  |
| Non-married |  | Reference |  |  | Reference |  |
| Married | 0.573 | 0.549-0.598 | <0.001 | 0.751 | 0.708-0.798 | <0.001 |
| Year of diagnosis | 0.885 | 0.881-0.889 | <0.001 | 1.006 | 0.999-1.013 | 0.102 |
| Chemotherapy |  |  |  |  |  |  |
| No |  | Reference |  |  | Reference |  |
| Yes | 0.343 | 0.128-0.921 | 0.034 | 2.000 | 0.366-10.925 | 0.423 |
| Radiation |  |  |  |  |  |  |
| No |  | Reference |  |  | Reference |  |
| Yes | 0.724 | 0.662-0.793 | <0.001 | 0.833 | 0.734-0.945 | 0.004 |
| Behavior behavior |  |  |  |  |  |  |
| Benign |  | Reference |  |  | Reference |  |
| Borderline malignancy | 0.764 | 0.697-0.836 | <0.001 | 0.886 | 0.783-1.002 | 0.055 |
| Surgery |  |  |  |  |  |  |
| No |  | Reference |  |  | Reference |  |
| Yes | 0.496 | 0.472-0.521 | <0.001 | 0.953 | 0.890-1.020 | 0.162 |
| Income |  |  |  |  |  |  |
| ≤35,000 |  | Reference |  |  | Reference |  |
| 35,000-75,000 | 0.877 | 0.737-1.043 | 0.139 | 0.943 | 0.738-1.204 | 0.638 |
| ≥75,000 | 0.749 | 0.628-0.893 | 0.001 | 0.910 | 0.710-1.166 | 0.457 |
| Tumor site |  |  |  |  |  |  |
| Endocrine gland |  | Reference |  |  | Reference |  |
| Brain exclude [nerve](file:///C:/Users/wrx/AppData/Local/Programs/baidu-translate-client/resources/app.asar/app.html#/#) and endocrine | 1.117 | 0.996-1.253 | 0.058 | 0.735 | 0.631-0.855 | <0.001 |
| Nervous system | 1.880 | 1.776-1.991 | <0.001 | 0.993 | 0.916-1.075 | 0.856 |
| Other | 0 | 0 | 0.999 | - | - |  |
| Tumor size | 1.0001 | 1.0001-1.0002 | 0.001 | 1.000 | 1.000-1.000 | 0.003 |
| Number of benign borderline tumors |  |  |  |  |  |  |
| 1 |  | Reference |  |  | Reference |  |
| >1 | 1.100 | 0.997-1.213 | 0.058 | 1.061 | 0.924-1.219 | 0.398 |
| Laterality |  |  |  |  |  |  |
| Left |  | Reference |  |  |  |  |
| Right | 0.991 | 0.939-1.045 | 0.731 | 1.002 | 0.93-1.079 | 0.966 |
| Bilateral | 1.253 | 1.151-1.364 | <0.001 | 1.182 | 1.048-1.333 | 0.006 |
| Other | 0.68 | 0.644-0.719 | <0.001 | 1.001 | 0.928-1.081 | 0.973 |

Note: *, p<0.05.

Supplementary Table 3 Univariate logistics regression analyses among patients with non-malignant of CNS receiving RT.

| Variables | Before Matching | | | After Matching | | |
| --- | --- | --- | --- | --- | --- | --- |
|  | Univariate analysis | | | Univariate analysis | | |
|  | OR | 95% CI | P | OR | 95% CI | P |
| Age of diagnosis (Years) | 1.085 | 1.077-1.093 | <0.001* | 0.992 | 0.982-1.002 | 0.102 |
| Race |  |  |  |  |  |  |
| Black |  | Reference |  |  | Reference |  |
| White | 0.737 | 0.569-0.956 | 0.022 | 0.699 | 0.473-1.033 | 0.073 |
| Asian or Pacific Islander | 0.441 | 0.288-0.674 | <0.001 | 0.735 | 0.394-1.372 | 0.334 |
| American Indian | 0.640 | 0.199-2.064 | 0.455 | 2.206 | 0.223-21.836 | 0.499 |
| Origin |  |  |  |  |  |  |
| Non-Hispanic |  | Reference |  |  | Reference |  |
| Hispanic | 0.570 | 0.416-0.781 | <0.001 | 0.292 | 0.200-0.426 | <0.001 |
| Sex |  |  |  |  |  |  |
| Male |  | Reference |  |  | Reference |  |
| Female | 0.733 | 0.614-0.875 | 0.001 | 0.701 | 0.543-0.905 | 0.006 |
| Marital status |  |  |  |  |  |  |
| Non-married |  | Reference |  |  | Reference |  |
| Married | 0.566 | 0.475-0.676 | <0.001 | 1.303 | 1.012-1.676 | 0.040 |
| Year of diagnosis |  |  |  |  |  |  |
| 2004-2007 | 11.292 | 7.406-17.217 | <0.001 | 1.997 | 1.167-3.420 | 0.012 |
| 2008-20111 | 6.848 | 4.454-10.527 | <0.001 | 1.793 | 1.038-3.096 | 0.036 |
| 2012-2015 | 3.109 | 1.965-4.919 | <0.001 | 1.503 | 0.837-2.697 | 0.172 |
| 2016-2019 |  | Reference |  |  | Reference |  |
| Chemotherapy |  |  |  |  |  |  |
| No |  | Reference |  |  | Reference |  |
| Yes | 0.336 | 0.047-2.410 | 0.278 | 0.499 | 0.045-5.521 | 0.571 |
| Behavior behavior |  |  |  |  |  |  |
| Benign |  | Reference |  |  | Reference |  |
| Borderline malignancy | 0.705 | 0.526-0.944 | 0.019 | 0.849 | 0.570-1.263 | 0.418 |
| Surgery |  |  |  |  |  |  |
| No |  | Reference |  |  | Reference |  |
| Yes | 0.642 | 0.528-0.779 | <0.001 | 0.619 | 0.475-0.807 | <0.001 |
| Income |  |  |  |  |  |  |
| ≤35,000 | 0.913 | 0.400-2.083 | 0.829 | 0.902 | 0.284-2.867 | 0.861 |
| 35,000-75,000 | 1.250 | 1.031-1.515 | 0.023 | 0.866 | 0.654-1.147 | 0.316 |
| ≥75,000 |  | Reference |  |  | Reference |  |
| Tumor site |  |  |  |  |  |  |
| Endocrine gland |  | Reference |  |  | Reference |  |
| Brain exclude [nerve](file:///C:/Users/wrx/AppData/Local/Programs/baidu-translate-client/resources/app.asar/app.html#/#) and endocrine | 0.796 | 0.484-1.310 | 0.369 | 0.917 | 0.466-2.020 | 0.936 |
| Nervous system | 0.767 | 0.601-0.980 | 0.034 | 0.843 | 0.589-1.205 | 0.348 |
| Other | 0 | 0 | 0.999 | - | - | - |
| Tumor size | 1.000 | 1.000-1.000 | 0.055 | 1.000 | 1.000-1.000 | 0.525 |
| Number of benign borderline tumors |  |  |  |  |  |  |
| 1 |  |  |  |  | Reference |  |
| >1 | 1.048 | 0.725-1.515 | 0.802 | 1.311 | 0.758-2.269 | 0.332 |
| Laterality |  |  |  |  |  |  |
| Left |  | Reference |  |  |  |  |
| Right | 1.022 | 0.828-1.261 | 0.839 | 1.042 | 0.774-1.403 | 0.786 |
| Bilateral | 1.478 | 1.021-2.138 | 0.038 | 1.411 | 0.814-2.445 | 0.22 |
| Other | 1.252 | 0.987-1.588 | 0.064 | 1.048 | 0.746-1.472 | 0.787 |

Note: *, p<0.05.
